# Active Commuting Throughout Adolescence and Central Fatness before Adulthood: Prospective Birth Cohort Study

**DOI:** 10.1371/journal.pone.0096634

**Published:** 2014-05-02

**Authors:** David Martinez-Gomez, Gregore I. Mielke, Ana M. Menezes, Helen Gonçalves, Fernando C. Barros, Pedro C. Hallal

**Affiliations:** 1 Department of Physical Education, Sports and Human Movement, Faculty of Teacher Training and Education, Autonomous University of Madrid, Madrid, Spain; 2 Post-Graduate Program in Epidemiology, Federal University of Pelotas, Pelotas, Rio Grande do Sul, Brazil; Québec Heart and Lung Institute, Canada

## Abstract

**Background:**

Active commuting is a good opportunity to accumulate physical activity (PA) across the lifespan that potentially might influence central body fat. We aimed to examine the prospective associations of active commuting at 11, 15 and 18 years of age with central body fat at 18 years.

**Methods:**

Participants were part of a large birth cohort study in Pelotas, Brazil (n = 3,649 participants). Active commuting, leisure-time PA and income were self-reported at 11, 15 and 18 years. Waist circumference and trunk fat mass were collected at 18 years with the use of a 3-dimensional photonic scanner and dual-energy X-ray absorptiometry, respectively.

**Results:**

Active commuting at 11 years was not prospectively associated with central body fat. However, we found that active commuting at 15 and 18 years were prospectively and cross-sectionally associated with central body fat variables, respectively, in boys but not in girls. Also, boys in the highest tertile of accumulated active commuting (i.e., average of active commuting at 11, 13 and 18 years) were associated with −2.09 cm (95%CI: −3.24; −0.94) of waist circumference and −1.11 kg (95%CI: −1.74; −0.48) of trunk fat mass compared to boys in the lowest tertile. Analyses on changes in tertiles of active commuting from 11 and 15 years to 18 years with central body fat variables at 18 years showed that boys who remained consistently in the highest tertile or moved to a higher tertile had lower levels of central body fat compared to those consistently in the lowest tertile.

**Conclusions:**

Active commuting throughout adolescence in boys, especially during middle and late adolescence, is associated with lower levels in central fatness before adulthood.

## Introduction

Excess central body fat is both a clinical and public health problem in adulthood [1]. Adults with an elevated central body fat will likely have more visceral, liver, and ectopic fat, and therefore, a greater risk for premature mortality and obesity-related metabolic disorders such as hypertension, type 2 diabetes and hypercholesterolemia [1]–[3]. Since central body fat tracks at high levels from childhood to adulthood [4], public health interventions must be developed from early ages. Physical activity (PA) has many benefits on health and one of the most accepted is its protective effect against obesity because of its role in energy balance [5], [6].

Active commuting is a good opportunity to accumulate PA and decrease prolonged sitting (i.e. sedentary behaviors) across the lifespan, which potentially might influence body fat as well as other health outcomes regardless of body fat [7]. The impact of active commuting, mainly active commuting to school, in childhood and adolescence on central body fat later in life may be yielded by several mechanisms. A first mechanism suggests that active commuting to school would have benefits in their current central body fat. To date, some studies examined this mechanism, but there is no convincing evidence to suggest that active commuting to school is associated with lower levels of central body fat in youth [7]–[10]. A second and unexplored mechanism would be that active commuting to school might be important whether it is accumulated over a long period of time to achieve their benefits on health. This fact would be particularly important during adolescence where there is large decreasing in PA [11], [12]. Finally, a third and another unexplored mechanism is based on compelling evidence that active commuting in adulthood is positively associated with central body fat and other health outcomes [13]–[17], so that young people who commuted to school may be likely of being active commuters in adulthood.

These unexplored mechanisms may provide new insights to support long-term public health initiatives for promoting active commuting. Hence, this study aimed to examine the prospective associations of active commuting at 11, 15 and 18 years of age with central body fat at young adulthood in a large birth cohort study from Brazil. We also investigated active commuting tracking from 11 and 15 years to 18 years.

## Materials and Methods

### Design and Participants

We used data from the 1993 Pelotas (Brazil) Birth Cohort Study; this is a longitudinal prospective study. Detailed information about the cohort methods has been published elsewhere [18], [19]. In brief, this birth cohort enrolled 5,249 of the 5,265 newborn children (99.7%) in the calendar year of 1993 in Pelotas, a city in Southern Brazil. In 2004, when they were aged 11 years, all participants were searched for follow-up, and 4,452 members of the original cohort were tracked. In 2008, when they were aged 15 years, all individuals were sought again, and 4,325 were followed up. In 2010, another attempt was made to follow-up all individuals when aged 18 years, and this was successful in 4,106 of them. Before being involved in the cohort, parents or guardians of all participants signed a written informed consent. The study protocol was approved by the Ethics Committee of the Medical School from the Federal University of Pelotas.

### Family Income

Family income at age 11, 15 and 18 years was calculated as the sum of the salaries of all household members in the previous month. Family income was expressed in the Brazilian currency (BRL; ISO code 4217), which is equivalent to ∼2 US dollars.

### Active Commuting

Active commuting (min/week) was self-reported. At 11 and 15 years, active commuting was limited to active commuting to or from school, whereas active commuting at 18 years included not only active commuting to or from (high) school but also any other options such as commuting to or from work, shopping and visit friends. Two questions were asked about the mode of commuting to or from school at 11 and 15 years: (i) “how do you usually travel to school?” and (ii) “how do you usually travel from school?” Response options for both questions were walking, biking, car/motorcycle, bus, and others. Next, we asked the duration of commuting to or from school, and the total time spent in active commuting was estimated by multiplying the daily duration of walking or biking to and from school by five –number of school days per week. At 18 years, frequency and duration of total commuting was asked separately for walking and biking. The total time spent in active commuting was calculated by summing the product of number of days and duration of commuting per day in both modes.

### Leisure Time PA

Leisure time PA (min/week) was assessed by two validated questionnaires [20], [21]. The questionnaire at 11 and 15 years comprised a list of physical activities typically practiced by children and adolescents in the region with the possibility to add additional activities not included in the initial list [20]. For each activity, participants were asked whether they had practiced it or not over the last 7 days. For all positive answers, participants were requested to report the number of days of practice and the mean duration of engagement per day. The time spent in leisure time PA was derived by multiplying the number of days where a given activity was reported by the average daily duration, and summing the values across different activities. At age 18 years, leisure time PA was calculated using the leisure-time section of the long version of the International PA Questionnaire (IPAQ) for adults [21]. Participants reported frequency and duration of engagement in walking, moderate-intensity PA and vigorous-intensity PA. Leisure time PA at 18 years was calculated as the sum of the time spent per week in each category as suggested in the guidelines for data processing and analysis of the IPAQ (www.ipaq.ki.se). Both PA questionnaires have shown a good reliability and adequate validity to assess PA in young people and adults from Brazil [20], [21].

### Anthropometry and Body Composition

Information on body weight and height were collected using standardized protocols. Body mass index was calculated as weight in kilograms divided by height in meters squared. Waist circumference (cm) was obtained with the use of a three-dimensional photonic scanner (3DPS; TC2 model, TC^2^, Cary, NC) [22]. All participants adopted a standardized position, using stabilizing handholds to maintain the position. Two measures were performed in each participant, and a third measurement was performed when differences between the two measurements were greater than 10 mm. Trunk fat mass (kg) at 18 years was obtained by Dual-energy X-ray absorptiometry (DXA; model Lunar Prodigi, GE Healthcare, Madison, WI). Participants remained lying in a supine position with legs and arms together and parallel to the body during scanning. The upper trunk was separated from the arms by a line from the axilla to the acromion. The lower trunk was separated from the legs by an oblique line through the femoral neck. DXA and 3DPS instruments were calibrated weekly by the research team. In both protocols, subjects used adequate clothes that were given specifically to attend the measurements.

### Statistical Analysis

A total of 3,820 individuals (72.8% of original cohort) had complete information on active commuting variables in the three weaves (11, 15 and 18 years). Of them, 351 participants were excluded due to lack of information on family income and leisure-time PA at any of the three measurements, and body composition at 18 years. Hence, all analyses were performed with 1,689 boys and 1,780 girls (*n* = 3,469; 66.1% of original cohort) with complete data available for the current study.

Descriptive characteristics are presented as means ± SD or percentages and differences between sexes were assessed by one-way analysis of variance and Pearson’s chi-squared test for continuous and categorical variables, respectively. Participants were classified according to their time spent in active commuting at 11, 15 and 18 years based on sex-specific tertiles (low, middle and high). In order to evaluate tracking of active commuting from 11 to 18 years and 15 to 18 years we used Spearman’s correlation coefficients. Differences in leisure time PA and family income by tertiles of active commuting in each age were assessed by one-way analysis of variance with Bonferroni’s adjustment for multiple comparisons.

For evaluating the prospective association between active commuting at 11 and 15 years and central body fat (waist circumference by 3DPS and trunk fat mass by DXA) at 18 years, we used linear regression controlling for leisure time PA and family income at baseline, and leisure time PA and family income at 18 years. We also examined the cross-sectional association between active commuting and central body fat at 18 years, including leisure time PA and family income as confounder variables. The association between accumulated active commuting at 11, 15 and 18 years (average of the three measurements) and central body fat at 18 years was examined by multiple lineal regression controlling for accumulated levels of leisure time PA and family income at 11, 15 and 18 years. Previously, to take into account that active commuting at 11 and 15 years was limited to commuting to school and more options of commuting could be reported at 18 years, the three measurements were transformed into Z-values (standardized value = value – mean/SD) before calculating the average of them. All linear trends were calculated using its respective continuous variable.

Finally, we examined differences in central body fat by changes in tertiles of active commuting (consistently low, decreasing, consistently middle, increasing, consistently high) from 11 to 18 years and 15 to 18 years by multiple linear regression adjusted for changes in leisure time PA and family income from baseline (11 or 15 years) to 18 years, and leisure-time PA and family income at 18 years. All analyses were conducted using STATA v.11 for Macintosh and statistical significance was set at *P*<0.05.

## Results

The descriptive characteristics of the sample are shown in Table 1. Boys were taller and heavier than girls, although there were no significant differences in BMI between the sexes. Regarding central body fat, girls had higher levels of trunk fat than boys, whereas boys had higher values of waist circumference than girls. Levels of family income, active commuting and leisure time PA were higher in boys than girls.

**Table 1:** Descriptive characteristics of the sample at age 18 years.

	All	Boys	Girls	*P*_for sex_
*n*	3469	1689	1780	
Height (cm)	167.2±9.2	173.8±6.7	161.0±6.5	<0.001
Weight (kg)	65.5±13.6	70.3±13.1	60.9±12.5	<0.001
Body mass index (kg/m^2^)	23.4±4.2	23.2±3.9	23.5±4.6	0.074
Waist circumference by 3DPS (cm)	83.0±9.7	84.2±9.6	81.8±9.6	<0.001
Trunk fat mass by DXA (kg)	8.9±5.5	6.8±5.2	10.9±5.1	<0.001
Family income (BRL/day)	69.7±99.8	73.9±102.4	65.7±97.0	0.015
Active commuting (min/week)	277.8±478.2	322.6±539.2	235.2±407.7	<0.001
Leisure time physical activity (min/week)	389.0±557.0	558.5±659.0	228.2±373.8	<0.001

Values are mean ± standard deviation. BRL: Brazilian real. DXA: Dual-energy X-ray absorptiometry. 3DPS: 3-dimension photonic scanner.

The proportion of active commuters at 11 and 15 and 18 years was 88.3%, 78.4% and 88.3% in boys, and 86.1%, 69.4% and 87.7% in girls, respectively. Tertile means, 95% confidence intervals, and ranges of active commuting at 11, 15 and 18 years are displayed in Table 2. Tracking of active commuting from 15 to 18 years (rho = 0.16 and 0.13, *P*<0.001 for boys and girls, respectively) was slightly stronger than tracking from 11 to 18 years (rho = 0.12 for boys and 0.08 for girls, *P*<0.001). There were significant differences (all *P*<0.01) in leisure time PA by tertiles of active commuting at 15 years only among girls and at 18 years in both sexes (Table 2). Also, there were significant differences in family income (all *P*<0.001) by tertiles of active commuting at all ages in both sexes (Table 2).

**Table 2:** Active commuting at 11, 15 and 18 yeast by tertile and its association with leisure time physical activity and family income.

		Active commuting (min/week)		Leisure-time physical activity (min/week)	Family income (BRL/day)
Tertile	*n*	Mean (95%CI)	Range	Mean (95%CI)	Mean (95%CI)
**11 years**					
Boys					
Low	710	30.1 (28.5; 31.7)	0 to 50	427.2 (389.8; 464.7)	55.6 (45.9; 65.4)
Middle	470	89.3 (88.0; 90.6)	55 to 100	421.5 (371.7; 471.4)	30.7 (24.4; 37.0)*
High	509	177.4 (172.6; 182.2)	105 to 450	443.5 (395.1; 492.0)	23.5 (21.8; 25.3)*
Girls					
Low	756	28.1 (26.5; 29.6)	0 to 50	244.0 (218.0; 270.0)	47.0 (41.82; 52.3)
Middle	442	89.1 (87.8; 90.4)	55 to 100	250.8 (208.2; 293.4)	26.3 (24.17; 28.5)*
High	582	183.3 (177.2; 189.4)	105 to 1000	270.1 (239.4; 300.9)	23.2 (21.60; 24.8)*
**15 years**					
Boys					
Low	842	24.6 (23.0; 26.1)	0 to 50	511.1 (474.0; 548.2)	53.5 (48.7; 58.3)
Middle	389	94.6 (93.5; 95.6)	60 to 100	515.3 (457.0; 573.6)	33.9 (30.3; 37.4)*
High	458	191.1 (185.1; 197.2)	110 to 600	538.7 (483.0; 594.4)	30.0 (26.8; 33.1)*
Girls					
Low	594	1.8 (1.3; 2.3)	0 to 25	174.4 (150.1; 198.7)	67.1 (59.3; 74.9)
	593	73.0 (71.0; 75.0)	30 to 100	195.5 (169.6; 221.5)	35.4 (31.5; 39.4)*
High	593	199.0 (192.9; 205.1)	110 to 675	235.0 (206.4; 263.6)*	28.2 (26.1; 30.3)*
**18 years**					
Boys					
Low	571	40.7 (37.7; 43.7)	0 to 100	441.2 (396.8; 485.7)	90.2 (79.9; 100.5)
Middle	560	174.8 (171.4; 178.2)	100 to 250	543.8 (495.5; 592.1)*	71.4 (62.8; 79.9)*
High	558	759.4 (696.0; 822.9)	250 to 7140	693.2 (626.6; 759.8)* ^,^ †	59.8 (54.2; 65.4)*
Girls					
Low	634	37.4 (34.8; 39.9)	0 to 90	182.2 (155.5; 209.0)	76.3 (66.0; 86.6)
Middle	640	152.4 (149.5; 155.4)	95 to 210	220.7 (193.7; 247.8)	63.4 (57.3; 69.5)
High	506	587.9 (532.7; 643.0)	210 to 9300	295.2 (257.9; 332.6)* ^,^ †	55.1 (50.2; 60.0)*

BRL: Brazilian real.

*Significantly different from the low tertile (all *P*<0.01).

† Significantly different from the middle tertile (all *P*<0.05).

Table 3 shows the associations of active commuting at 11, 15 and 18 years with central body fat at 18 years. Active commuting at 11 years was not prospectively associated with central body fat in both sexes (all *P* for trend>0.05). However, we found that active commuting at 15 and 18 years were prospectively and cross-sectionally associated with central body fat variables in boys (all *P* for trend≤0.001) but not in girls (all *P* for trend>0.05). When analyzing differences in central body fat at 18 years between commuters and non-commuters participants at 11, 15 and 18 years, we found similar sex- and age-specific findings than previous analyses using tertiles (data not shown).

**Table 3:** Association of active commuting at 11, 15 and 18 years with central body fat at 18 years.

Tertile	*n*	Waist circumference by 3DPS (cm)	Trunk fat mass by DXA (kg)
**11 years** a			
Boys			
Low	710	Reference	Reference
Middle	470	−0.87 (−2.01; 0.23)	−0.55 (−1.16; 0.06)
High	509	−0.30 (−1.40; 0.79)	−0.29 (−0.98; 0.31)
* P*_for trend_		0.211	0.097
Girls			
Low	756	Reference	Reference
Middle	442	0.39 (−0.76; 1.53)	0.43 (−0.17; 1.03)
High	582	0.25 (−0.81; 1.31)	0.14 (−0.42; 0.70)
* P*_for trend_		0.446	0.895
**15 years** b			
Boys			
Low	842	Reference	Reference
Middle	389	−1.07 (−2.22; 0.08)	−0.49 (−1.11; 0.14)
High	458	−1.60 (−2.70; −0.50)*	−0.82 (−1.41; −0.22)*
* P*_for trend_		<0.001	0.001
Girls			
Low	594	Reference	Reference
Middle	593	1.34 (0.22; 2.45)*	0.17 (−0.42; 0.76)
High	593	1.12 (−0.2; 2.25)	0.20 (−0.41; 0.80)
* P*_for trend_		0.073	0.568
**18 years**			
Boys			
Low	571	Reference	Reference
Middle	560	−0.67 (−1.78; 0.44)	−0.36 (−0.97; 0.25)
High	558	−2.28 (−3.41; −1.15)*	−1.22 (−1.83; −0.61)*
* P*_for trend_		<0.001	<0.001
Girls			
Low	634	Reference	Reference
Middle	640	0.50 (−0.55; 1.56)	0.23 (−0.33; 0.78)
High	506	0.23 (−0.91; 1.35)	0.06 (−0.53; 0.66)
* P*_for trend_		0.290	0.133

Values are mean (95% confidence interval). DXA: Dual-energy X-ray absorptiometry. 3DPS: 3-dimension photonic scanner Analyses were adjusted for leisure time physical activity and family income at 18 years.

a Additionally adjusted for leisure time physical activity and family income at 11 years.

b Additionally adjusted for leisure time physical activity and family income at 15 years.

*Significantly different from the low tertile (all *P*<0.05).

**Figure 1:**
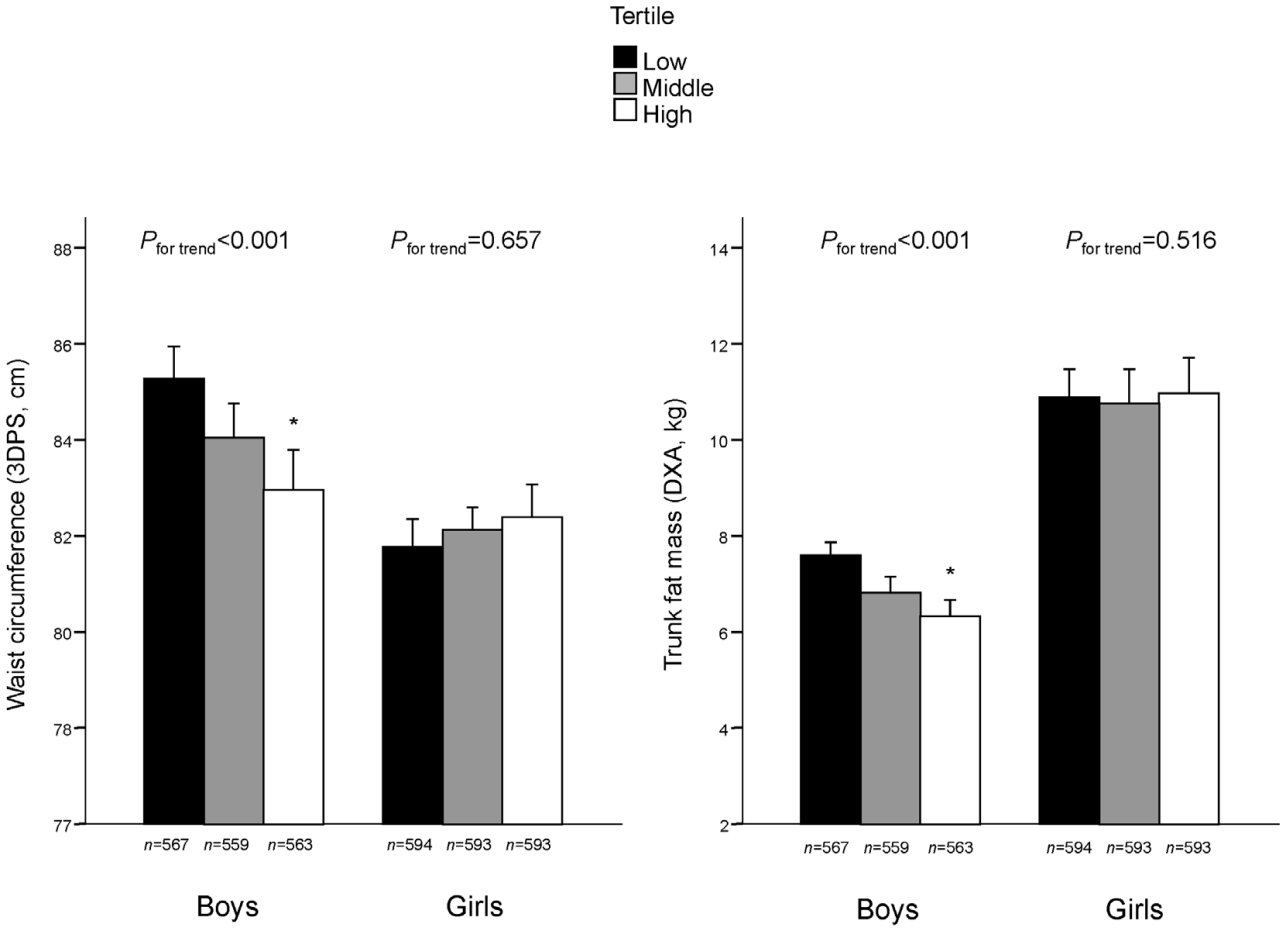
Association between accumulated active commuting at 11, 15 and 18 years and central body fat at 18 years. Footnotes: Values are mean (95% confidence interval). DXA: Dual-energy X-ray absorptiometry. 3DPS: 3-dimension photonic scanner. Accumulated active commuting at 11, 15 and 18 years was calculated as the average of the three measurements (min/week). To take into account that active commuting at 11 and 15 years was limited to commuting to school and more options of commuting could be reported at 18 years, the three measurements were transformed into Z-values (standardized value = value – mean/SD) before calculating the average of them. Linear trends were calculated using continuous variable. Analyses were adjusted for accumulated changes in leisure time physical activity and family income at 11, 15 and 18 years. *Significantly different from the low tertile (all *P*<0.05).

The association of accumulated active commuting at 11, 15 and 18 years was also significantly associated (all *P* for trend <0.001) with both central body fat variables only in boys (Figure 1). After controlling for accumulated levels of leisure time PA and family income, boys in the highest tertile of active commuting were associated with −2.09 cm (95%CI: −3.24; −0.94) of waist circumference and −1.11 kg (95%CI: −1.74; −0.48) of trunk fat mass compared to boys in the lowest tertile.

Lastly, we examined the association of changes in tertiles of active commuting from 11 and 15 years to 18 years with central body fat variables at 18 years. Overall, boys who remained consistently in the highest tertile or moved to a higher tertile from 11 and 15 years to 18 years had lower levels of central body fat compared to those consistently in the lowest tertile (Table 4). Yet, boys who remained consistently in the middle tertile was only associated with lower levels of trunk fat mass from 11 to 18 years. (Table 4) In addition, boys with a decreasing pattern in active commuting from 11 to 18 years were no associated with central body fat variables, but from 15 to 18 years did it, so that boys who moved to a lower tertile had lower levels of waist circumference and trunk fat compared to those consistently in the lowest tertile (Table 4).

**Table 4:** Changes in active commuting from 11 and 15 years to 18 years and central body fat at 18 years.

Changes in tertile	*n*	Waist circumference by 3DPS (cm)	Trunk fat mass by DXA (kg)
**11 to 18 years** a			
Boys			
Consistently low	266	Reference	Reference
Decreasing	475	−0.64 (−2.09; 0.80)	−0.43 (−1.32; 0.36)
Consistently middle	152	−1.78 (−3.69; 0.12)	−1.11 (−2.15; −0.06)*
Increasing	614	−2.16 (−3.55; −0.77)*	−1.22 (−1.97; −0.50)*
Consistently high	182	−2.92 (−4.75; −1.10)*	−1.74 (−2.74; −0.75)*
Girls			
Consistently low	301	Reference	Reference
Decreasing	554	−0.26 (−1.63; 1.12)	0.09 (−0.71; 0.73)
Consistently middle	165	0.62 (−1.22; 2.45)	0.62 (−0.34; 1.59)
Increasing	591	−0.08 (−1.43; 1.26)	0.08 (−0.70; 0.72)
Consistently high	369	0.52 (−1.32; 2.36)	0.36 (−0.61; 1.33)
**15 to 18 years** b			
Boys			
Consistently low	332	Reference	Reference
Decreasing	395	−2.33 (−3.74; −0.92)*	−1.30 (−2.27; −0.53)*
Consistently middle	125	−1.42 (−3.38; 0.54)	−0.70 (−1.77; 0.36)
Increasing	652	−1.89 (−3.22; −0.56)*	−1.05 (1.78; −0.33)*
Consistently high	185	−3.68 (−5.50; −1.86)*	−2.01 (−3.00; −1.02)*
Girls			
Consistently low	256	Reference	Reference
Decreasing	607	1.18 (−0.27; 2.62)	0.19 (−0.64; −0.88)
Consistently middle	210	1.94 (0.17; 3.71)*	0.45 (−0.48; 1.39)
Increasing	516	0.91 (−0.59; 2.42)	0.30 (−0.49; 1.09)
Consistently high	191	1.66 (−0.23; 3.55)	0.32 (−0.67; 1.32)

Values are mean (95% confidence interval). DXA: Dual-energy X-ray absorptiometry. 3DPS: 3-dimension photonic scanner Analyses were adjusted for leisure time physical activity and family income at 18 years.

a Additionally adjusted for changes in leisure time physical activity and family income from 11 to 18 years.

b Additionally adjusted for changes in leisure time physical activity and family income from 15 to 18 years.

*Significantly different from the low tertile (all *P*<0.05).

Since waist circumference and body mass index were strongly related in our study (rho = 0.88 and 0.87 in boys and girls), we reanalyzed our data and similar association patterns were found between active commuting and body mass index. Also, when entering body mass index as a covariate in all analyses with waist circumference as the outcome, the main results did not change (data not shown).

## Discussion

In this birth cohort study from Brazil, our main findings indicate that being engaged in active commuting throughout adolescence, especially at high levels, is associated with lower levels of central body fat at late adolescence in boys but not in girls. Importantly, we found that the closer to adulthood, the stronger the benefits of active commuting on central fatness in boys. Since we also found that active commuting track at low levels from 11 and 15 years to 18 years, these results support long-term public health initiatives to increase and maintain high levels of active commuting from early ages to adulthood to fight against excess central body fat in boys.

Central obesity is a worldwide known risk factor, and consequently, one of the main drivers of chronic diseases and premature deaths [1]–[3]. Low PA might be a potential contributor in the pathogenesis of excess central body fat throughout life [5], [6]. Since a child with high levels of central fat mass is more likely to become an adult with excess central fatness [4], it highlights the need of public health interventions to increase PA as early as possible.

Although active commuting to school is a daily opportunity to increase PA in youth, recent systematic reviews indicate that there is no compelling evidence for an association with central obesity [8]–[10]. Previous studies with cross-sectional data found inconsistent results. For example, in a Canadian sample with 315 children (mean age of 10.1 years) there was no association between active commuting to school and waist circumference [23]. Conversely, in a similar sample, Pizarro et al. [24] found that among 229 Portuguese children (mean age of 11.7 years), those who walked to school had higher odds of having a <90^th^ waist circumference. To date, a few studies with longitudinal data and interventions exist in the scientific literature [8]–[10] but most of them examined the association of active commuting to school with total body fat (e.g. body mass index, skinfolds). Two longitudinal studies [25], [26] and one clinical trial [27] that included measurements of central body fat were conducted in North European populations with unusual large proportions of children and adolescences biking to school. Consequently and owing to the small sample sizes included, these studies examined changes in central body fat between youth who usually bicycling to school and those who use other modes, including walking. Yet, a potential greater effect of cycling could be attributed to higher intensity during bicycling compared to walking, the three studies found no significant associations between changes in bicycling to school and changes in waist circumference [25]–[27].

Most of the evidence aforementioned suggests, therefore, that active commuting to school is not associated with central fatness in childhood and adolescence, but it could be still important for preventing adult excess central body fat. Our results extend previous research by examining whether accumulated active commuting over a long period, that is, throughout adolescence, is associated with central body fat before adulthood. However, it is worth keeping in mind that we found similar results with body mass index, that is, a typical measure of total body fat, in additional analyses. We found that continued high levels of active commuting throughout adolescence is associated with lower levels of central body fat at late adolescence. Hence, the earlier start to engage in high levels of active commuting, the lower its detrimental impact on central fatness before adulthood. However, this relationship was observed in boys but not in girls. We found this circumstance in both cross-sectional and prospective analyses, which strengthens a plausible sex-specific effect.

A possible explanation would be that active commuting in boys is performed at higher intensities than girls. Thus, the preferred walking speed in boys could be at moderate or even high intensities (>1 m/s), whereas girls preferred walking speed at light-intensity (≤1 m/s). We cannot confirm this issue in this study but in additional analyses we also found that boys were more likely to use the bicycle as compared to girls in three measurements (6.8% vs. 2.0% at 11 years, 10.1% vs. 1.6% at 15 years, and 45.7% vs. 22.1% at 18 years, respectively). Therefore, active commuting in girls would not accumulate to meet the PA guidelines for youth of 60 minutes per day in moderate-to-vigorous PA, which are associated with healthy levels of fat [6]. Future research should to investigate what factors are associated with PA intensities during active commuting throughout adolescence.

Another important finding in our study was that the impact of active commuting on central fatness in boys was stronger, the closer we get to adulthood. For example, boys in the highest tertile of active commuting at 11, 15 and 18 year were associated with −0.3 cm, −1.6 cm, and −2.3 cm in waist circumference compared to those in the lowest tertile, respectively. Our results stress, therefore, that in boys each passing year in adolescence without engagement in high levels of active commuting may be critical for his future central obesity. However, we found that tracking of active commuting was low (r<0.2). Taken together, such information support to develop sustainable and effective interventions to participate and increase levels of active commuting throughout adolescence in boys, particularly in middle and late adolescence.

Our results in a relatively large birth cohort also indicate that adolescents in low-income families engage in lower levels of active commuting than their peers in middle- and high-income families. This finding suggests, hence, that adolescents in families with better socioeconomic status must be target populations for interventions designed to increase levels of active commuting. On the other hand, since Brazil is nowadays a developing country with large inequalities, it partially might explain the high proportion of adolescents who are active commuters in our sample. The prevalence of active commuters in this Brazilian cohort was greater than most developed countries such as Australia, Spain, UK, USA, and Portugal, but similar than in some North-European countries (i.e. Norway and Denmark) with long cultural tradition of active commuting [7]–[9], [28]–[33].

Some of the strengths of the present work are the relative large sample size from a birth cohort, its prospective design, the inclusion of important confounders (i.e. leisure time PA and family income), precise and accurate clinical assessments of central adiposity, and the substantial time interval between measurements that include the entire adolescence. This study has limitations as well. Changes in the main outcomes were not available in previous waives. This fact limits the ability to draw conclusions with regard to the real impact of active commuting on central fatness development. Although we can assume that central fatness have certain stability over time [4], some changes are still possible and would likely have led to an underestimation of the beneficial influence of active commuting with central body fat. On the other hand, PA measures were self-reported (mode and duration of commuting and leisure time PA); therefore, our results must be interpreted with caution. Future studies using isolated or combined objective tools to assess active commuting (e.g. online route planners, global positioning system, geographic information system, accelerometry) and its intensity may provide further evidence on these relationships. Also, other measures of PA in school settings (e.g. recess and physical education) could be not controlled in our analyses. Finally, the low prevalence of cycling to school (4–5%) in our sample hampers to perform the analyses separately for walkers and cyclists due to statistical power limitations.

In summary, our results suggest that active commuting throughout adolescence is associated with lower levels of central body fat before adulthood in boys but not in girls. Additionally, we found that the benefits of active commuting on central fatness were stronger as they approached adulthood. Taken into consideration that active commuting tracks at low levels, public health strategies for promoting active modes of commuting throughout adolescence seems to be important to close the ‘age gap’ on central fatness levels among boys.
